# Risk and severity of SARS-CoV-2 reinfection among patients with multiple sclerosis vs. the general population: a population-based study

**DOI:** 10.1186/s12883-022-02907-8

**Published:** 2022-10-08

**Authors:** Mahdi Barzegar, Amirreza Manteghinejad, Sara Bagherieh, Setayesh Sindarreh, Omid Mirmosayyeb, Shaghayegh Haghjooy Javanmard, Vahid Shaygannejad, Maryam Nasirian

**Affiliations:** 1 Department of Neurology, School of Medicine, Isfahan University of Medical Sciences, Isfahan, Iran; 2 Isfahan Neurosciences Research Center, Isfahan University of Medical Sciences, Isfahan, Iran; 3 Cancer Prevention Research Center, Omid Hospital, Isfahan University of Medical Sciences, Isfahan, Iran; 4 Department of Biostatistics and Epidemiology, Faculty of Health, Isfahan University of Medical Sciences, Isfahan, Iran; 5 Applied Physiology Research Center, Cardiovascular Research Institute, Isfahan University of Medical Sciences, Isfahan, Iran

**Keywords:** Multiple sclerosis, COVID-19, SARS-CoV-2, Reinfection, Rituximab

## Abstract

**Background:**

We conducted this study to compare the risk of reinfection between multiple sclerosis (MS) patients and a control group without MS.

**Method:**

In this retrospective study, data of all SARS-CoV-2 tests (*n* = 793,301) and almost all MS patients (*n* = 10,639) in Isfahan province were collected from January 01, 2020 to August 22, 2021. Of the 2196 MS patients and 793,301 persons from the general population who had been tested at least once, 3 control for each MS patient were identified, leaving 1560 MS patients and 4680 controls without MS. We compared the risk of reinfection after 90 days of a primary infection between those with and without a previous positive COVID-19 test.

**Results:**

736 (47.2%) MS patients and 2013 (43.0%) control individuals had at least one positive test. A total of 17 (2.3%) and 22 (1.1%) possible reinfections in MS and control groups were observed. The estimated protection against reinfection in all MS patients, MS patients on rituximab, MS patients on DMTs rather than rituximab, and controls were 68.2% (46.2, 81.2%), 57.4% (− 0.1, 83.1%), 71.5% (45.5, 85.2%), and 82.1% (72.1, 88.5%), respectively. We found no statistically significant difference in estimated protection (*p* = 0.123) and odd of reinfection (adjusted OR: 2.01 [0.98, 4.08]) between all MS patients and control group. Two patients were hospitalized at first infection but none required hospitalization at reinfection event.

**Conclusions:**

MS patients on rituximab may be at a greater risk of reinfection. Further studies are required to assess the risk of the second reinfection among the MS population.

## Introduction

As coronavirus disease (COVID-19) pandemic continues, a question regarding herd immunity after the disease and the risk of reinfection has been raised. There is an urgent need to better understand whether those who have recovered from COVID − 19 are secure from reinfection. Because the effectiveness of vaccination strategies and general modeling for the epidemic depend on the effectiveness and period of immunity against COVID-19.

Most cases of reinfection were observed among people with immunosuppressive therapies and elderly individuals [1, 2]. Reports of reinfection in immunocompromised patients increased concern regarding multiple sclerosis (MS) patients since they are mostly on immunosuppressant agents. Some attempts have investigated the SARS-CoV-2 antibody response in MS patients [3–5]. They showed a decreased antibody response in MS patients who were treated with anti-CD20 therapies. These findings strengthen the assumption of an increased risk of reinfection in these patients. Therefore, there is an urgent need to estimate the reinfection rate in MS patients.

The province of Isfahan, Iran, has experienced five waves of COVID-19 with more than 300,000 laboratory-confirmed cases. In this context, we carried out this study to compare the risk of reinfection among MS patients with a non-MS control group from the Isfahan general population in the setting of a population-based prospectively collected data of all cases who have been tested for SARS-CoV-2 in Isfahan province.

## Method

### Data collection and study design

In response to the COVID-19 outbreak in Iran, Isfahan University of Medical Science launched Isfahan COVID-19 Registry (I-CORE) to collect data of all individuals who were tested for COVID-19 with a residential address in Isfahan province, except those are in the city of Kashan [6]. Because Kashan University of Medical Sciences independently registered persons with COVID-19 in the city of Kashan. The I-CORE registry includes individual-level data on all SARS-CoV-2 polymerase chain reactions (PCR), rapid antigen tests, hospitalization, and death. Rapid antigen testing became available in Iran in December 2020. After that, most suspected persons were tested by rapid antigen test. The data of SARS-CoV-2 PCR and rapid antigen test were collected regardless of the reason for the test and COVID-19-related symptoms. Data on hospitalization includes all individuals with a suspect or confirmed COVID-19 diagnosis who needed hospitalization. Almost all hospitalized patients were tested for SARS-CoV-2. We also collected data on individuals who died of any cause during the study from the dataset of the community health center.

The Vice-Chancellery for Clinical Affairs provides medical and social support for MS patients. Their dataset coverages near all confirmed MS patients who are residents in the Isfahan province (*n* = 10,639), except those who are living in the city of Kashan. The information includes the date of birth, sex, date of MS diagnosis, course of MS (clinically isolated syndrome [CIS], relapsing-remitting MS [RRMS] and progressive MS [PMS], and disease-modifying therapy (DMT). All data were extracted on September 1–3, 2021, and were linked together using the national identification number. Foreign residents were excluded from the study.

We conducted this study as part of the study of COVID-19 susceptibility and outcome among the Isfahan MS population. The study was approved by the regional bioethics committee of Isfahan University of Medical Sciences (IR.MUI.MED.REC.1400.391).

### Reinfection definition

We included all individuals in the Isfahan province who underwent SARS-CoV-2 PCR or rapid antigen test during the study. Individuals with a PCR or rapid antigen test, regardless of the results, were followed-up till the end of the study, whether through the date of reinfection or death. Subjects with a positive SARS-CoV-2 test (SARS-CoV-2 PCR or rapid antigen tests) were considered as the infected group. A positive SARS-CoV-2 test (PCR or rapid antigen test) after 90 days of the initial one was considered as possible reinfection [7, 8]. Persons with an initially negative test were defined as the uninfected group. A positive test after 90 days of a negative one changes the subject’s situation from uninfected to the infected group. Individuals with an initial negative test who became positive within 90 days counted switched to the infected group and counted as a unique case.

Due to the absence of information on viral RNA sequencing, the probability of reinfection was evaluated clinically. Our method was broadly based on the method of Leidi and colleagues [9] to estimate the protection against reinfection among the Sweden general population. A clinician telephonically interviewed MS patients with suspected reinfection to rule out other respiratory infections and protracted RNA detection. Adjudication, if available, was done based on the reason for testing, developing COVID-19 related symptoms, history of contact with a confirmed/suspected COVID-19 case, history of positive household contact, PCR cycle threshold (Ct), and report of computerized tomography (CT) scan. COVID-19-related symptoms were fever, cough, shortness of breath, and anosmia or dysgeusia [10, 11].

### Statistical analysis

For each MS patient who was tested for SARS-CoV-2, 3 individuals with at least one test were randomly selected from the general population. We calculated the incidence person time as patients with at least one positive divided by the time interval from the beginning of follow-up to the first positive SARS-CoV-2 test or the end of follow-up. We estimated the risk ratios (RR) with 95% confidence intervals (95%CI) of being positive for SARS-CoV-2, comparing patients who were previously positive and negative using Poisson regression analysis. Infection protection against reinfection was calculated as (1 – adjusted RR) × 100 [2]. We also estimated the odds ratios (OR) and their 95% CI of reinfection among individuals with a previous positive test comparing the MS and control groups using logistic regression analysis. The RR and OR were also calculated for MS patients on rituximab and those who were treated with all other DMTs. For MS patients, the model was adjusted for age, sex, receiving a COVID-19 vaccine, MS course, and MS duration. The model was adjusted for age, sex, and receiving a COVID-19 vaccine for the general population. We performed two sensitivity analyses, one in which participants had at least two SARS-CoV-2 tests and one in which subjects had at least three SARS-CoV-2 tests. We performed all statistical analysis using Stata software (version 14, Stata Corporation, College Station, Texas, USA). A *P*-value less than 0.05 was considered as significant.

## Results

**Fig. 1:**
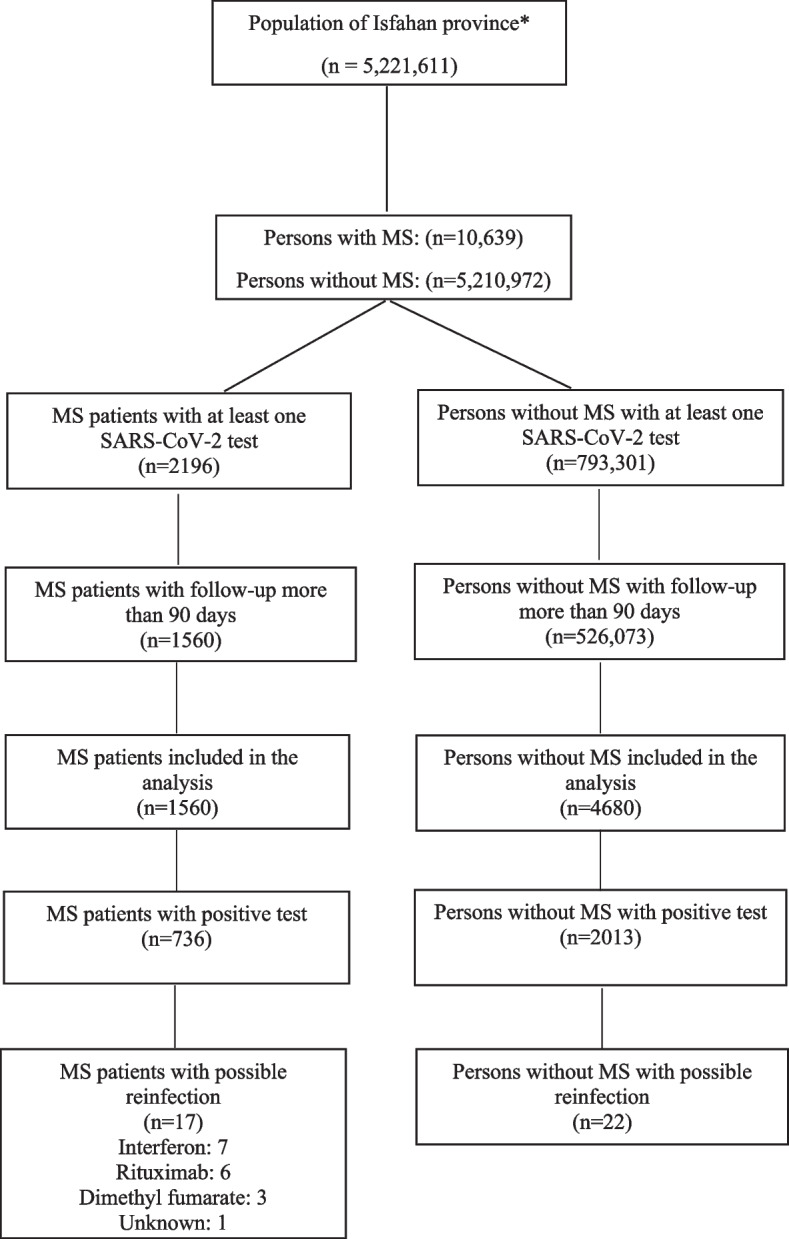
Flowchart of the study. ^*^Population of the Isfahan province in 2021, except those who were resident in the city of Kashan

During the follow-up, 2196 MS patients and 793,301 of the general population were tested for COVID-19 at least once. Six hundred and thirty-six MS patients and 60,000 individuals from the general population were excluded due to a follow-up duration of fewer than 90 days. Of the remaining, we randomly identify 3 individuals from the general population, leaving 1560 MS patients and 4680 control individuals without MS. The flowchart of the study is shown in Fig. 1. Characteristics of these subjects are shown in Table 1. For the MS patients, the mean age was 39.19 (9.7), 1144 (73.3%) were female, and 1298 (83.2%) were RRMS. The most common DMTs was interferon (579, 37.1%), following by rituximab (358, 23.0%), dimethyl fumarate (187, 11.8%), and glatiramer acetate (129, 8.3%).

**Table 1:** Characteristics of participants included in the study

	Multiple Sclerosis			Control group			***P***-Value^**1**^
	Total *n* = 1560	Conversion from negative to positive *n* = 74	Reinfection *n* = 17	Total *n* = 4680	Conversion from negative to positive *n* = 191	Reinfection *n* = 22	
Age, years, Mean (SD)	39.19 (9.7)	35.0(8.38)	37.7(8.9)	42.92(18.2)	45.720.7)	44.0(16.9)	< 0.0001
Sex, female, n (%)	1144 (73.3)	58(78.4)	13(76.5)	2146 (45.9)	79(41.4)	13(59.1)	< 0.0001
Number of tests^2^, n (%)							
One	1203(77.1)	0(0)	0(0)	3675(78.5)	0(0)	0(0)	0.243
Two	276(17.7)	46(62.2)	12(70.6)	760(16.24)	129(67.5)	11(50)	0.182
More than two	81(5.2)	28(37.8)	5(29.4)	245(5.2)	62(32.5)	11(50)	0.948
Initial positive test^2^, n (%)	627(40.2)	0(0)	14(82.4)	1728(36.9)	0(0)	19(86.4)	0.021
Initial negative test^2^, n (%)	933(59.8)	74(100)	3(17.7)	2952(63.1)	191(100)	3(13.6)	
Switch^2^, n (%)	35(3.7)	0(0)	2(11.8)	94(3.2)	0(0)	3(13.6)	0.956
At least one positive test^2^ during follow-up-n (%)	736(47.2)	74(100)	17(100)	2013(43.0)	191(100)	22(100)	0.004
At least one positive PCR test during follow-up-n (%)	679 (43.5)	47 (63.5)	1 (5.9)	1746 (37.3)	112 (58.6)	6 (27.3)	< 0.0001
Follow-up duration^3^, days Mean(SD)	257.6 (109.7)	231.5(10.3.2)	205.6(84.9)	244.6(112.6)	217.5(103.2)	241.9(99.6)	0.001
Incidence Person-time×10^4^(95% CI)^4^	18.3 (17.0, 19.7)	3.2 (2.5, 4.0)	1.0 (0.6, 1.5)	17.6 (16.8, 18.4)	2.7 (2.3, 3.1)	0.5 (0.3, 0.7)	0.345
Vaccine type							
BBIBP-CorV	919 (58.9)	22 (29.7)	8 (47.1)	836 (17.9)	16 (8.4)	4 (18.2)	< 0.0001
ChAdOx1 nCoV-19	47 (3.0)	1 (1.3)	1 (5.9)	435 (9.3)	6 (3.1)	4 (18.2)	
Gam-COVID-Vac	39 (2.5)	3 (4.0)	0(0)	65 (1.4)	1 (0.5)	0(0)	
Covaxin	5 (0.3)	1 (1.3)	0(0)	18 (0.4)	3 (1.6)	0(0)	
COVIran Barekat	14 (0.9)	0(0)	0(0)	96 (2.1)	2 (1.0)	0(0)	
At least one dose of a vaccine, n (%)	1024 (65.6)	27(36.5)	9 (5.9)	1450 (31.0)	28(14.7)	8 (36.3)	< 0.0001
Second dose of a vaccine, n (%)	823 (52.8)	20(27.0)	7 (41.1)	523 (11.2)	10(5.2)	2(9.09)	< 0.0001
Fully immunized^5,6^, n (%)	800 (51.3)	18(24.3)	2 (11.8)	365 (7.8)	10(5.2)	1 (4.5)	< 0.0001
MS type, n (%)							
RRMS	1298 (83.2)	67(90.5)	14(82.4)	–	–	–	–
SPMS	68 (4.4)	2(2.7)	0(0)	–	–	–	–
CIS	74 (4.8)	1(1.4)	1(5.9)	–	–	–	–
Unknown	120(7.7)	7(5.4)	2(11.8)	–	–	–	–
MS Duration, year- Mean (SD)	7.78 (5.64)	6.16(4.7)	7.5(4.5)	–	–	–	–
DMTs							
Interferon	579 (37.1)	26(35.1)	7(41.2)	–	–	–	–
Glatiramer acetate	129 (8.3)	4(5.4)	0(0)	–	–	–	–
Fingolimod	114 (7.3)	7(9.5)	0(0)	–	–	–	–
Dimethyl fumarate	187 (11.8)	6(8.1)	3(17.7)	–	–	–	–
Teriflunomide	85 (5.5)	7(9.5)	0(0)	–	–	–	–
Rituximab	358 (23.0)	20(27.0)	6(35.3)	–	–	–	–
Natalizumab	18 (1.2)	0(0)	0(0)	–	–	–	–
Other therapy	62 (4.0)	3(4.1)	0(0)	–	–	–	–
Unknown	28(1.8)	1(1.35)	1(5.88)	–	–	–	–

^1^Comparison between all MS patients and all control individuals is significant if *P*-value< 0.05

^2^PCR or Rapid-Antigen

^3^Follow-up duration was computed from including in the study through the time of being positive, dead or ended of study, whichever occurred first

^4^Being positive at least once during the follow-up

^5^Participants were considered fully immunized 14 days after receiving second dose of a COVID-19 vaccine

^6^Six participants (3 MS patient and 3 person in general population) became re-infected after vaccination

The mean of follow-up in MS and control groups were 257.6 (109.7) and 244.6 (112.6) days, respectively. The test density incidence per 10,000 persons during follow-up in MS and control groups were 18.3 (95%CI: 17.0, 19.7) and 17.6 (95%CI: 16.8, 18.4), respectively (*p* = 0.345). Six hundred and twenty-seven (40.2%) MS patients and 1728 (36.9%) of the control group had an initial positive test. Thirty-five MS patients and 94 control subjects with initial negative tests switched to the infected group. Seventy-four MS and 191 control individuals converted from uninfected to the infected group. In total, 736 (47.2%) MS patients and 2013 (43.0%) control individuals had at least one positive test.

During follow-up, there were 17 (2.3%) and 22 (1.1%) possible reinfections in MS and control groups. The characteristics of MS patients with suspected reinfection are summarized in Table 2. Seven patients were on interferon, 6 were on rituximab, and 3 were on dimethyl fumarate. Fourteen patients were RRMS, and one was CIS. Two MS patients were hospitalized after the initial infection, but none required hospitalization following the second infection. All MS patients with reinfection recovered completely after the primary infection and were asymptomatic during the follow-up. All patients but one developed COVID-19-related symptoms at the second infection. All MS patients with reinfection reported a history of contact with a suspected/confirmed case. Out of 17 MS patients who had reinfection, eight received BBIBP-CorV and one received ChAdOx1 nCoV-1. Three patients developed reinfection after the second dose of vaccines. Of 22 control individuals with reinfection, eight received SARS-CoV-2 vaccines (BBIBP-CorV: 4, ChAdOx1 nCoV-19: 4) during the study. Of whom, two developed reinfections after the first dose of ChAdOx1 nCoV-19 and one who had received BBIBP-CorV developed reinfection after the second dose of vaccine.

**Table 2:** Characteristics of MS patients with possible reinfection

Characteristics						First Spiecemen				Second spiecmen			Third spiecemen			Interval between first and second positive tests	Vaccine status	
	Age	Sex	Disease duration	Course of MS	DMT	Number of tests	Date of test	COVID-19 symptoms	Hositalization	Date of test	COVID-19 symptoms	Hositalization	Date of test	COVID-19 symptoms	Hositalization		First dose	Second dose
1	33	F	10	RRMS	DMF	3	23.05.2020	Present	No	28.03.2021^a^	Absence	No	05.04.2021	Present	No	317	–	–
2	39	M	14	RRMS	DMF	3	29.08.2020^a^	Present	No	13.09.2020	Present	No	0.9.04.2021	Present	No	208	06.05.2021	03.06.2021
3	37	F	9	RRMS	DMF	3	27.10.2020	Present	No	05.03.2021	Present	No	29.08.2021^a^	Present	No	129	03.05.2021	02.06.2021
4^c^	36	F	7	Uknown	Interferon	3	10.08.2020^a^	Absence	No	13.02.2021	Present	No	12.08.2021	Present	No	180	26.04.2021	30.07.2021
5	40	F	8	RRMS	Interferon	3	01.11.2020^a^	Present	No	06.12.2020	Present	No	15.05.2021	Present	No	160	22.06.2021	–
6	12	M	1	RRMS	Rituximab	2	15.08.2020	Present	No	14.04.2021	Present	No	–	–	No	242	–	–
7	26	M	3	RRMS	Rituximab	2	15.05.2021	Present	Yes^b^	05.09.2021	Present	No	–	–	No	113	–	–
8	31	F	8	RRMS	Interferon	2	14.04.2021	Present	No	14.09.2021	Present	No	–	–	No	153	–	–
9	30	F	5	RRMS	Interferon	2	29.09.2020	Present	No	12.01.2021	Present	No	–	–	No	105	05.05.2021	02.06.2021
10	30	M	3	CIS	Uknown	2	27.07.2020	Present	No	12.04.2021	Present	No	–	–	No	259	–	–
11	33	F	6	RRMS	Rituximab	2	07.10.2020	Present	No	11.09.2021	Present	No	–	–	No	339	–	–
12^c^	44	F	17	RRMS	Rituximab	2	22.05.2021	Absence	No	30.08.2021	Present	No	–	–	No	100	02.05.2021	03.07.2021
13^c^	37	F	9	Uknown	Interferon	2	04.09.2020	Present	No	12.09.2021	Present	No	–	–	No	373	03.05.2021	02.06.2021
14	44	F	14	RRMS	Interferon	2	22.10.2020	Present	Yes	21.02.2021	Present	No	–	–	No	122	03.05.2021	31.05.2021
15	37	F	3	RRMS	Rituximab	2	09.09.2020	Present	No	04.04.2021	Present	No	–	–	No	207	22.06.2021	–
16	36	F	1	RRMS	Interferon	2	11.02.2021	Present	No	15.09.2021	Present	No	–	–	No	216	–	–
17	50	F	9	RRMS	Rituximab	2	08.11.2020	Present	No	08.08.2021	Absence^d^	No	–	–	No	273	–	–

All vaccinated patients received BBIBP-CorV, except case 4 received ChAdOx1 nCoV-19

*RRMS* relapsing-remitting multiple sclerosis, *CIS* clinically isolated syndrome, *DMF* dimethyl fumarate

^a^Negative result. Other tests were positive

^b^Needed supplementary oxygen therapy and ventilation

^c^Cases 12 and 13 developed reinfection after full immunization and case 4 had reinfection 13 days after the second dose of ChAdOx1 nCoV-19

^d^Patients developed non-specific symptoms including fatigue and myalgia

The adjusted RR of infection among previously infected patients compared to uninfected persons in MS and control individuals were 0.318 (95%CI: 0.188, 0.538) and 0.179 (95%CI: 0.115, 0.279) (Table 3). The estimated protection against reinfection in MS and control were 68.2% (95%CI: 46.2, 81.2%) and 82.1% (95%CI: 72.1, 88.5%). No statistically significant difference in estimated protection between MS and the control group was found (*p* = 0.123). The rate of positive tests was significantly lower in MS patients on rituximab who had a previous positive test than those who had previously only tested negative (crude RR: 0.388, 95%CI: 0.156, 0.967). However, the difference did not remain statistically significant after adjustment (adjusted RR: 0.426, 95%CI: 0.169, 1.070). The adjusted RR of infection in MS patients on other DMTs was 0.285 (95% CI: 0.148, 0.545). The protection against reinfection in MS patients with rituximab and those on other DMTs were 57.4% (95%CI: − 0.1, 83.1%) and 71.5% (95%CI: 45.5, 85.2%). No significant difference in protection between MS patients on rituximab compared to those on other DMTs (*p* = 0.235) was observed.

**Table 3:** Rate of infection and reinfection in MS patients vs. control individuals

	n	Conversion from negative to positive	Infection Person-days follow-up	Infection rate× 10^4^ (95% CI)	Reinfection	Reinfection Person-days follow-up	Reinfection rate× 10^4^ (95% CI)	Crude Rate Ratio of reinfection (96% CI)	Adjusted Rate Ratio reinfection (96% CI)	*P*-value	Estimated Protection% (96% CI)	Crude Odds Ratio (96% CI)	Adjusted Odds Ratio(96% CI)
**Main analysis of reinfection during the study**													
MS + Rituximab	358	20	52,760	3.79 (2.44,5.86)	6	40,885	1.46 (0.66,3.20)	0.388 (0.156,0.967)*	0.426 (0.169,1.070)^a^	0.235	57.4 (−0.1,83.1)	1.08(0.65,4.94)^c^	1.92(0.69,5.37) ^a,c^
MS + other DMTs	1202	54	177,151	3.04 (2.33,3.98)	11	131,163	0.83 (0.46,1.50)	0.280 (0.146,0.537)*	0.285 (0.148,0.545) ^*a^		71.5 (45.5,85.2)*		
All MS patients	1560	74	229,911	3.21 (2.56,4.04)	17	172,018	0.98 (0.61,1.50)	0.312 (0.183,0.527)*	0.318 (0.188,0.538)*^b^	0.123	68.2 (46.2,81.2)*	2.15(1.37,4.08)^*d^	2.01(0.98,4.08) ^b,d^
Control group	4680	191	691,489	2.76 (2.39,3.18)	22	453,435	0.48 (0.32,0.73)	0.181 (0.116,0.281)*	0.179 (0.115,0.279)*^b^		82.1 (72.1,88.5)*		
**Sensitivity analysis of reinfection in people with at least two SARS-CoV-2 tests**													
MS + Rituximab	72	20	14,519	13.77 (8.88,21.35)	6	11,401	5.26 (2.36,11.7)	0.357 (0.143,0.889)*	0.380 (0.151,0.952)*^a^	0.046*	62.0 (4.80,84.9)*	1.15 (0.39,3.15)^c^	1.12 (0.37,3.38)^a,c^
MS + other DMTs	204	54	47,086	11.46 (8.78,14.97)	11	22,971	4.78 (2.65,8.64)	0.416 (0.217,0.797)*	0.451 (0.231,0.875)*^a^		54.9 (12.5,76.9)*		
All MS patients	276	74	61,605	12.01 (9.56,15.08)	22	34,372	4.94 (3.08,7.96)	0.406 (0.239,0.687)*	0.402 (0.237,0.682)*^b^	0.042*	59.8 (31.8,76.3)*	2.24 (1.14,4.37)*^d^	2.09 (0.99,4.41)^b,d^
Control group	760	191	166,286	11.48 (9.96,13.23)	17	93,231	2.35 (1.55,3.58)	0.218 (0..140,0.339)*	0.220 (0.141,0.343)*^b^		78.0 (65.7,85.9)*		
**Sensitivity analysis of reinfection in people with at least three SARS-CoV-2 tests**													
MS + Rituximab	20	9	4092	21.99 (11.44,42.27)	0	1640	–	–	–	–	–	–	–
MS + other DMTs	61	19	11,588	16.39 (10.45,25.70)	5	4204	11.89 (4.95–28.57)	0.740 (0.276–1.97)	0.882 (0.398,2.52)^a^		11.8 (−48.0,39.7)		
All MS patients	81	28	15,680	17.85 (12.32,25.86)	5	5844	8.55 (3.56,20.55)	0.510 (0.197,1.32)	0.512 (0.198,1.33)^b^	0.332	48.8 (−67.0,80.2)	1.90 (0.57,6.25)^d^	1.23 (0.32,4.74)^b,d^
Control group	245	62	43,018	14.41 (11.23,18.48)	11	22,081	4.98 (2.75,8.99)	0.379 (0.200,0.721)	0.399 (0.209–0.761)*^b^		60.1 (23.9,79.1)*		

^*^Significant (*P* value < 0.05)

^a^Adjusted for gender, age, MS type, Covid,19 vaccination, and MS duration

^b^Adjusted for gender, age, Covid,19 vaccination

^c^The odds ratio of Rituximab in reinfection

^d^The odds ratio of MS in reinfection

MS patients were more likely to have reinfection after a positive test compared to the control group (OR = 2.15, 95%CI: 1.37, 4.08). However, this did not remain significant after adjustment (OR = 2.01, 95%CI: 0.98, 4.08). There was no statistically significant difference between rituximab and other DMTs in the odds of reinfection in both adjusted and unadjusted models (unadjusted: 1.08, 95%CI: 0.65, 4.94; adjusted OR: 1.92, 95%CI: 0.69, 5.37).

In two sensitivity analyses, we restricted our sample to the people with at least two and at least three SARS-CoV-2 tests (Table 3). In people with at least two tests, there was a significant difference in the rate of positive tests in MS patients on rituximab who had a previous positive test compared to those who had previously only tested negative (adjusted RR: 0.380, 95%CI: 0.151,0.952). No further change in the direction and significance of RR and OR was observed. Because of small sample size, we were unable to estimate protection against reinfection in MS patients on rituximab who had at least three SARS-CoV-2 tests.

## Discussion

Finding the risk of SARS-CoV2 reinfection among previously infected persons is crucial for understanding the herd immunity after the infection and optimizing vaccination programs. However, it is still not known whether these patients are more prone to develop reinfection. We found protection against a second infection in the MS population to be 68.2%. We observed a non-significant increased odd of reinfection in MS patients than control group.

The protection against reinfection in our control group from the general population was 82.1%. Comparable results have been reported in previous studies. A large population-based study using Danish national surveillance dataset of 4 million PCR-tested individuals found an 80.5% decreased risk of repeat infection [2]. Another prospective cohort study of health-worker in the UK found that patients with a previous history of COVID-19 were 84% protected against reinfection [10]. A retrospective study in the USA showed that a previous history of COVID-19 was associated with an 81.8% decreased risk of second reinfection [12]. A recent meta-analysis of 15 studies estimated the protection against reinfection as 87.1%, with 95% confidence interval between 82.4 and 90.6% [13].

To explore the effect of DMTs on the risk of reinfection, we examined the reinfection rate by DMTs. The protection in MS patients on rituximab decreased to 57.4%. There was no significant difference in the rate of positive tests in MS patients on rituximab who had a previous positive test compared to those with no detectable SARS-CoV-2 in previous tests. This result is supported by a recent study from England that compared the incidence risk ratio of SARS-CoV-2 infection between MS patients and the general population before and after mass vaccination. They found an increase in infection risk in MS patients treated with ocrelizumab, as an anti-CD20 agent, compared to the general population after vaccination [14]. A multi-center retrospective study from Italy showed a higher rate of SARS-CoV-2 mRNA Vaccine breakthrough infection in fully vaccinated MS patients on ocrelizumab and fingolimod than in patients treated with other DMTs [15].

The possible increased risk of reinfection and breakthrough SARS-CoV-2 infections after vaccination might be related to waned humoral response in MS patients on anti-CD20 therapies. In such cases, an undetectable SARS-CoV-2 antibody in infected MS patients with anti-CD20 was observed [16–18]. Further studies indicated a decreased rate of seroconversion and titer of IgG SARS-CoV-2 antibody in MS patients with B cell depletion therapies than other DMTs and control groups [3, 19–21]. The observed poor humoral response is attributed to these agents’ mechanism of action, which eliminates B-cells subsets, including pre-B cells, naive B cells, and memory B cells that express CD20 [22]. An important point to bear in mind is the role of T-cells and neutralizing antibodies in protection from reinfection [23–25]. Studies showed that MS patients treated with anti-CD20 agents generated robust SARS-CoV-2-specific T-cell responses following SARS-CoV-2 infection in the absence of pronounced humoral response [26, 27]. However, the measure of immunity is provided with T-cell response to COVID-19 in MS patients treated with anti-CD20 agents is unclear.

Epidemiological and non-human studies showed that previous SARS-CoV-2 infection is associated with protection against severe reinfection [28–31]. However, it is not clear whether the severity of reinfection in MS patients is greater or milder than the primary infection. In our study, none of MS patients with reinfection needed hospitalization. These results suggest that severity of reinfection is not severe than the primary infection. There is a reason to believe that the severity of reinfection in MS patients could be similar or milder than the first infection. The current evidence suggests that even low level of neutralizing antibody following COVID-19 can protect patients against severe reinfection [31]. Moreover, cellular response is associated with less severe reinfection [32, 33]. Therefore, poor acquired humoral and cellular immunity of a previous infection in MS patients may limit the severity of reinfection. However, because reinfection was rare and most patients were on safe DMTs, further investigations should seek to explore the severity of reinfection among the MS population.

This study has limitations that should be considered. The lack of sequence information of the virus genomes was the main limitation. Therefore, we cannot confirm whether the reinfections result from prolonged viral shedding or new infection. Our database included data on all SARS-CoV-2 tests without knowing the reason for testing. We lack information on socioeconomic status, comorbidity, and other treatments of MS and the general population, which could have affected the risk of reinfection. Our study is limited by a lack of data on COVID-19 treatment such as monoclonal antibodies or antiviral treatments, which can affect the outcome of COVID-19 infection. The small number of reinfection among MS samples did not allow us to compare the risk of repeat infection by each DMTs. People with underlying medical conditions, especially MS, who are at greater risk of severe infection, may have more tendency for SARS-CoV-2 testing following respiratory symptoms. Having a prior positive test also may actuate people for more testing. As a result, an overestimation of reinfection may be possible. It is noteworthy that this overestimation affects both groups. It should be noted that 5 MS patients with reinfection were tested three times, and others were tested only twice. Moreover, the number of tests and the test density incidence between MS and control group was similar. This shows that the increased risk of reinfection among MS patients may not be related to repeated tests. The information on the coronavirus variant was not documented. Therefore, we were unable to assess the risk of reinfection in different coronavirus variants. False negative SARS-CoV-2 tests in hospitalized patients is another limitation. One of the sources of limitation is that PCR and rapid antigen tests were performed using different commercial assays. Because of the difference in sensitivity and specificity between SARS-CoV-2 PCR and the rapid antigen test, we investigated the reinfection rate among those who underwent only the PCR test. Due to the small number of reinfection (one MS patient and six control individuals), we could not estimate the reinfection rate in patients who underwent only PCR tests. The reason for the increasing use of rapid antigen throughout the study is that this test became available in December 2020. Since then, rapid antigen has been more easily accessible than PCR tests. Because of small sample size, the results of sensitivity analysis of reinfection in people with at least three SARS-CoV-2 tests should be treated with causation. The reason for the difference in the coverage of vaccination between MS and the control group is that MS patients were among the first groups who received the COVID-19 vaccine in Iran. A reasonable explanation for a high percentage of BBIBP-CorV in MS patients is that the Isfahan MS society recommended BBIBP-CorV over other SARS-CoV-2 vaccines. We believe that our study design is not appropriate to assess the impact of vaccination against SARS-CoV-2 reinfection. Therefore, we should avoid drawing any conclusion about the effect of vaccination on the risk of new infection and reinfection.

Our study has some strengths. A main strength of the present study includes using a population-based database with coverage of entire people in Isfahan province who underwent PCR or rapid antigen test and the patients required hospitalization. This reduces the selection bias. Another strength is long follow-up time up to more than 8 months. We clinically adjudicate the probability of reinfection clinically. The symptoms of all patients were completely resolved at frist infection. Moreover, all but one developed COVID-19-realted symptoms at reinfection. This strength the probability of true reinfection in our MS patients.

To our knowledge, this is the first observational study to estimate the risk of reinfection among the MS population. Understanding the strength and durability of immunity following the COVID-19 infection in MS patients treated with immunosuppressive agents has been a major question. Our result suggests that MS patients with rituximab may be at greater risk of reinfection. Further studies are needed to investigate the risk of reinfection among MS patients.

## Data Availability

The datasets used and/or analysed during the current study available from the corresponding author on reasonable request.
